# Body composition and changes in health-related quality of life in older age: a 10-year follow-up of the Helsinki Birth Cohort Study

**DOI:** 10.1007/s11136-020-02453-1

**Published:** 2020-03-02

**Authors:** Tuija M. Mikkola, Hannu Kautiainen, Mikaela B. von Bonsdorff, Minna K. Salonen, Niko Wasenius, Eero Kajantie, Johan G. Eriksson

**Affiliations:** 1 Folkhälsan Research Center, Helsinki, Finland; 2 Clinicum, Faculty of Medicine, University of Helsinki, Helsinki, Finland; 3 Primary Health Care Unit, Kuopio University Hospital, Kuopio, Finland; 4 Gerontology Research Center, Faculty of Sport and Health Sciences, University of Jyväskylä, Jyvaskyla, Finland; 5 Public Health Promotion Unit, National Institute for Health and Welfare, Helsinki, Finland; 6 Department of General Practice and Primary Health Care, University of Helsinki and Helsinki University Hospital, Helsinki, Finland; 7 PEDEGO Research Unit, MRC Oulu, Oulu University Hospital and University of Oulu, Oulu, Finland; 8 Department of Clinical and Molecular Medicine, Norwegian University for Science and Technology, Trondheim, Norway; 9 Children’s Hospital, Helsinki University Hospital and University of Helsinki, Helsinki, Finland; 10 Singapore Institute for Clinical Sciences, Agency for Science, Technology, and Research, Singapore, Singapore; 11 Department of Obstetrics & Gynaecology, Yong Loo Lin School of Medicine, National University of Singapore, Singapore, Singapore

**Keywords:** Health-related quality of life, Aging, Body composition, Obesity, Lean mass, Fat mass

## Abstract

**Purpose:**

Most studies examining the associations between body composition and health-related quality of life (HRQoL) in older age have been cross-sectional and analyzed only fat or lean mass. Hence, it is poorly known whether fat and lean mass are independently associated with subsequent changes in HRQoL. We investigated whether baseline lean and fat mass are associated with changes in HRQoL over a 10-year period in older adults.

**Methods:**

We studied 1044 men and women from the Helsinki Birth Cohort Study (age 57–70 years at baseline). Bioelectrical impedance analysis was used to derive baseline fat mass index (FMI, fat mass/height^2^) and lean mass index (lean mass/height^2^), dichotomized at sex-specific medians. HRQoL was assessed using RAND 36-item Health Survey at baseline and follow-up 10 years later.

**Results:**

When controlled for lean mass and adjusted for potential confounders, high baseline FMI was associated with a greater decline in general health (standardized regression coefficient [*β*] = − 0.13, *p* = 0.001), physical functioning (*β* = − 0.11, *p* = 0.002), role physical (*β* = − 0.13, *p* = 0.003), vitality (*β* = − 0.08, *p* = 0.027), role emotional (*β* = − 0.12, *p* = 0.007), and physical component score (*β* = − 0.14, *p* < 0.001). High baseline FMI was also associated with low HRQoL in all physical domains at baseline (*β*: from − 0.38 to − 0.10). Lean mass was not strongly associated with HRQoL at baseline or change in HRQoL.

**Conclusion:**

In older community-dwelling adults, higher fat mass is, independent of lean mass, associated with lower physical HRQoL and greater decline in HRQoL. Prevention of adiposity may contribute to preservation of a good quality of life in older age.

## Introduction

Obesity is globally a growing health concern among older adults, and it is associated with a number of physical health problems in older age [1–3]. It increases the risk of many non-communicable diseases, such as osteoarthritis, cardiovascular disease (CVD), and type 2 diabetes [4], which may further lead to reduction in health-related quality of life (HRQoL). Excess fat may contribute to the development of mobility limitations [5] subsequently leading to decrease in physical HRQoL, since mobility ability is an essential component of physical HRQoL. Lean mass, which is mostly composed of skeletal muscle tissue, has health implications although its impact on health appear to be less striking than that of fat mass in the general population [6]. However, the importance of lean mass is pronounced in older age. Muscle mass tends to decrease with aging [7] and may eventually reach a critical threshold where its level is too low for managing daily activities independently [8]. Lean mass can be used as a marker of nutritional status and it has been reported to be associated with length of stay in hospital [9]. Sarcopenia, that is low muscle mass co-occurring with low muscle strength or low physical performance [10], has been reported to be associated with worse overall HRQoL in older adults [11]. However, not all studies support this finding [12].

A number of studies have examined the associations between obesity, based on BMI, and HRQoL among older adults [13–16]. Overweight and obesity have been reported to be associated with poorer overall HRQoL [16] and physical HRQoL [13–15] but not with mental HRQoL [14, 15]. However, although BMI is a useful measure of overweight and obesity in the general adult population, predicting many health indicators [4, 17], it is less applicable to older adults [18, 19]. Body composition undergoes marked changes with aging; there is a loss in muscle mass, while the proportion of fat tissue increases and fat is redistributed in the body [20–22] making body composition of an average older adult very different from that of an average younger adult. Furthermore, irrespective of age, there is a large variation in the relative proportions of fat and lean mass among persons with the same BMI. This calls for use of more accurate methods to quantify body composition and body compartments in studies focusing upon older adults. Most previous studies investigating the associations between body compartments and HRQoL in older adults have examined either fat or lean mass and have not taken into account their mutual effects [23–25]. This may cause confounding bias as fat and lean mass are closely correlated; those with high fat mass tend to also have high lean mass. To avoid confounding in the analysis, it is essential to include both fat mass and lean mass in the same analysis to yield their independent effects and also their possible interactions.

A vast majority of previous studies have analyzed the associations of fat and lean mass with HRQoL in cross-sectional settings and only few studies have assessed whether body composition is associated with change in HRQoL in older age [26, 27]. Hence, the purpose of this study was to examine cross-sectional associations of body composition with physical and mental HRQoL and to assess whether baseline body composition is associated with subsequent change in HRQoL during a 10-year period among older adults.

## Methods

### Sample

The Helsinki Birth Cohort Study (HBCS) consists of 13,345 singletons, who were born in Helsinki in 1934–1944 and were still alive in 1971. A total of 8760 individuals were born in the Helsinki University Central Hospital and of these people, 2902 individuals were randomly selected to participate in a clinical examination in 2001–2004 to reach a target of 2000 participants. In total, 2003 individuals participated in the baseline clinical examination. The age range of the participants was 57–70 years (mean 61 years) at baseline. Follow-up examinations were performed in 2011–2013 when the participants were 67–79 years old (mean 71 years) (*n* = 1094). Of these participants, body composition was not obtained from 39 participants at baseline, and they were excluded from the analysis. Further, two participants did not return HRQoL questionnaire at baseline, and nine did not return HRQoL questionnaire at follow-up and were, therefore, excluded. Hence, 1044 participants with complete data on body composition at baseline and HRQoL at both measurement points were included in the present analysis. The study design and assessments during the clinical visits have been described in detail previously [28–30]. All measurements were performed by trained study nurses.

### Body composition and anthropometry

Body composition was assessed at baseline by bioelectrical impedance analysis using the InBody 3.0 eight-polar tactile electrode system (Biospace Co, Ltd, Seoul, Korea) [31]. The instrument estimates lean body mass and body fat mass by segmental multi-frequency (5, 50, 250, and 500 kHz) analysis. The measurements were made with the subject standing in light indoor clothing on the four foot electrodes on the platform of the analyzer and gripping the two palm and thumb electrodes. Between-day precision of InBody 3.0 has been reported to be 2.7% [31]. When compared to DXA fat-free mass, percent root mean square error of InBody 3.0 was 6% [31]. Fat and lean mass indices were calculated as follows: fat mass index (FMI, kg/m^2^) = fat mass/height^2^ and lean mass index (LMI, kg/m^2^) = lean mass/height^2^.

Height and weight were measured in light indoor clothing and without shoes. Height was measured to the nearest 0.1 cm using a Kawi stadiometer and weight to the nearest 0.1 kg using Seca Alpha 770 scales. Body mass index (BMI) was calculated as weight in kilograms divided by the square of height in meters. Waist circumference was measured midway between the lowest rib and the iliac crest twice using a soft tape. The average of the two measurements was calculated.

### Health-related quality of life

Health-related quality of life was assessed using the Finnish validated version of the RAND 36-Item Heath Survey version 1.0 questionnaire [32]. The RAND-36 is composed of eight domains: physical functioning (10 items), role limitations caused by physical health problems (4 items), bodily pain (2 items), general health (5 items), role limitations caused by emotional problems (3 items), vitality (4 items), mental health (5 items) and social functioning (2 items). The single items in the questionnaire were coded to range between 0 and 100, with 100 representing the best level of functioning or well-being. Domain scores were the averages of the items. Domain score was set as missing if more than half of the items in a given domain was missing. Physical (PCS) and mental health summary scores (MCS) were aggregated from the eight domain scores. First, these domains were standardized using the means and standard deviations of the US reference population (1990) [33]. Next, the domains were weighted using factor score coefficients obtained from the same reference population and summed to yield PCS and MCS. Finally, PCS and MCS were standardized using a mean of 50 and a standard deviation of 10 [34]. The sub-scales of RAND-36 have high unidimensionality and sufficiently high internal consistency among older adults, which makes RAND-36 a useful measure in studies of older adults [35].

### Socioeconomic variables

Date of birth was retrieved from hospital birth records and was used to calculate subjects’ age at the start of the follow-up. Years of education were derived from information on the level of education obtained from the nation-wide register of Statistics Finland. Income (corresponding to value in euros in 2018) per consumption unit in 2000 was calculated as follows: household taxable income divided by the square root of the number of people in the household [36]. These data were obtained from Statistics Finland and were linked using unique personal identification codes.

### Long-term illness

Information on severe long-term diseases was extracted from the national Care Register for Health Care (former Hospital Discharge Register) for the time period between Jan 1st 1971 and June 30th 2000 and was linked using unique personal identification codes. The register contains information on all specialized outpatient visits (from 1998 onwards) and hospital inpatient discharges in Finland. From the register, we aimed to identify severe long-term illnesses, which seriously compromise physical or mental functioning of the subject. Severe long-term illnesses from the following disease categories were included: cancer, cardiovascular disease, pulmonary, dementia, psychiatric, neurological, musculoskeletal, kidney, gastrointestinal, and diabetes. Any severe long-term illness variable was coded as 1 (severe long-term illness) and 0 (no severe long-term illness).

### Lifestyle variables, blood pressure, and blood lipids

Information on lifestyle was obtained using a questionnaire administered at baseline (2001–2004). The participants were asked about their marital status, smoking, and alcohol consumption. Marital status and smoking were dichotomized (cohabiting yes/no, current smoker yes/no). Frequency of alcohol consumption was recoded into four categories (0/1–2 times per month/1–2 times per week/ > 2 times per week). The participants also completed a validated Kuopio Ischemic Heart Disease Risk Factor Study (KIHD) questionnaire to assess the duration, frequency and intensity of leisure-time physical activity during the past 12 months [37]. For each intensity grade, activity-specific metabolic equivalent (MET) values were used. Total leisure-time physical activity, including both non-conditioning (e.g., housework) and conditioning (e.g., resistance training) physical activity, was computed and is expressed in METhours per day.

Blood pressure was measured from the right arm while the subject was in a sitting position and was recorded as the mean of two successive readings from a mercury sphygmomanometer. Blood samples were drawn for the assessment of glucose and lipids. Serum cholesterol and triglyceride concentrations were measured with the use of standard enzymatic methods. LDL cholesterol concentrations were calculated using the Friedewald formula [38].

### Statistical analysis

Fat and lean mass indices were divided according to median values, separately for men and women, after which four body composition categories were created: (1) low FMI and low LMI (LFLL), (2) low FMI and high LMI (LFHL), (3) high FMI and low LMI (HFLL), (4) high FMI and high LMI (HFHL). By dividing participants into these categories, we aimed at reducing confounding between fat and lean mass indices. Median values for FMI were 17.8 kg/m^2^ in women and 6.0 kg/m^2^ in men and for LMI 8.84 kg/m^2^ in women and 20.8 kg/m^2^ in men, respectively. The associations of body composition categories with continuous background characteristics and HRQoL measures were analyzed using general linear models. The associations of body composition categories with categorical background characteristics were analyzed using (ordinal) logistic regression analysis. In the cross-sectional linear regression models with HRQoL measures as dependent variables, continuous FMI and LMI and their interaction were entered into the models as independent variables. In the adjusted models, sex, age, education, smoking, alcohol consumption, physical activity, and any severe long-term illness were set as covariates because they potentially affect both body composition and HRQoL. Changes in HRQoL measures during the follow-up were calculated as the absolute differences between the follow-up values and corresponding baseline values. In the longitudinal regression models, changes in HRQoL measures were first regressed on continuous baseline FMI and LMI and their interaction. These models were adjusted for the same covariates as the cross-sectional analyses and additionally for the corresponding baseline HRQoL domain scores. These cross-sectional and longitudinal linear regression analyses were repeated with dichotomized baseline FMI and LMI and their interaction as independent variables. Predictive margins were computed for the interaction term to yield adjusted predictions of HRQoL means for the four body composition categories. Bootstrapping with 5000 repetitions was used to calculate 95% confidence intervals. Standardized regression coefficients from models using continuous FMI and LMI as independent variables were used as effect size indicators. The values of the standardized regression coefficients above 0.10, 0.30, and 0.50 represent small, moderate and large effect sizes, respectively [39]. The data were analyzed using Stata 15.1 (StataCorp, College Station, TX, USA).

## Results

**Fig. 1:**
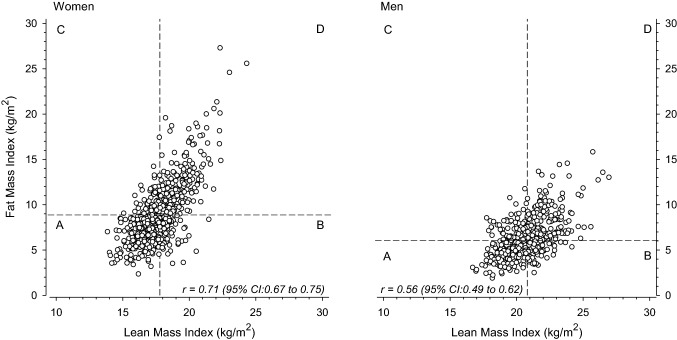
Relationships between fat mass index and lean mass index in women (left panel) and men (right panel). Fat and lean mass indices were each divided according to sex-specific median values into two categories and based on them, four categories were created: a lean low fat low, b lean high fat low, c lean low fat high, d lean high fat high

The relationships between lean mass index and fat mass index and the median-split categories of body composition in women and men are shown in Fig. 1.

Compared to those included in the analysis, the subjects who took part only in the baseline measurements, and hence were excluded from the present study, were older (mean 61.4 vs. 60.7 years, *p* < 0.001) and less educated (11.0 vs. 11.7 years, *p* < 0.001). The excluded cohort members were more frequently men (49% vs. 43%, *p* = 0.008) and more likely to have a high fat mass index (55% vs. 46%, *p* < 0.001) compared to cohort members who were included in the present study. However, there was an equal proportion of those with a high lean mass index among the included and excluded subjects (50% vs. 50%, *p* = 0.91).

### Background characteristics

Table 1 shows the background characteristics of the subjects grouped according to the four body composition categories derived based on dichotomized fat mass index and lean mass index. The four body composition categories (LFLL, LFHL, HFLL, HFHL) did not differ with respect to sex, income, marital status, smoking, physical activity, total cholesterol concentration, or prevalence of severe long-term illnesses other than diabetes. Age was somewhat lower in the LFHL category than in the other categories (*p* = 0.054). LDL cholesterol concentration was lowest in the LFLL category and highest in the LFHL category (*p* = 0.063). Waist circumference, BMI, blood pressure, and triglycerides increased with increasing fat and lean mass index, while HDL cholesterol concentration decreased with increasing fat and lean mass index. The proportion of individuals with diabetes was highest in the HFHL category.

**Table 1:** Background characteristics of the Helsinki Birth Cohort Study participants in 2001–2004

	Fat mass index low			Fat mass index high		*p*-value^a^
	Lean mass index low *N* = 377	Lean mass index high *N* = 144		Lean mass index low *N* = 144	Lean mass index high *N* = 379	
Women, *n* (%)	222 (59)	73 (51)		73 (51)	223 (59)	0.13
Age, years mean (SD)	60.6 (2.7)	60.3 (2.2)		61.1 (3.0)	60.9 (2.9)	0.054
Education years, mean (SD)	12.0 (3.7)	12.3 (3.1)		11.6 (3.6)	11.1 (3.3)	< 0.001
Income in 1000 euros, mean (SD)	60.5 (48.8)	56.1 (34.1)		55.2 (66.4)	53.3 (47.8)	0.25
Cohabiting, *n* (%)	290 (77)	112 (78)		105 (73)	301 (79)	0.46
Current smoker, *n* (%)	69 (18)	35 (24)		31 (22)	64 (17)	0.22
Alcohol consumption, *n* (%)						< 0.001
Not at all	24 (6)	1 (1)		10 (7)	21 (6)	
1–2 times/month	139 (37)	54 (38)		48 (34)	179 (48)	
1–2 times/week	151 (40)	52 (37)		53 (37)	130 (35)	
> 2 times/week	62 (16)	35 (25)		32 (22)	46 (12)	
Daily physical activity in METhours, mean (SD)	5.6 (3.6)	5.9 (4.1)		4.8 (3.2)	5.3 (3.8)	0.085
Waist circumference in cm, mean (SD)	85 (9)	91 (7)		96 (8)	104 (11)	< 0.001
BMI in kg/m^2^, mean (SD)	23.4 (1.8)	26.1 (1.4)		27.4 (1.6)	31.4 (3.5)	< 0.001
Blood pressure in mmHg						
Systolic, mean (SD)	139 (19)	141 (19)		145 (22)	149 (19)	< 0.001
Diastolic, mean (SD)	85 (10)	87 (10)		90 (9)	91 (10)	< 0.001
Total cholesterol in mmol/l, mean (SD)	5.82 (0.94)	5.94 (0.92)		5.93 (1.15)	5.91 (1.11)	0.48
LDL in mmol/l, mean (SD)	3.53 (0.80)	3.73 (0.83)		3.66 (0.99)	3.63 (0.89)	0.063
HDL in mmol/l, mean (SD)	1.76 (0.45)	1.66 (0.44)		1.60 (0.41)	1.51 (0.40)	< 0.001
Triglycerides in mmol/l, mean (SD)	1.20 (0.57)	1.22 (0.52)		1.47 (0.60)	1.70 (0.86)	< 0.001
Severe long-term illnesses, *n* (%)						
Cardiovascular	28 (7)	7 (5)		8 (6)	28 (7)	0.65
Pulmonary	5 (1)	2 (1)		1 (1)	5 (1)	0.96
Psychiatric	7 (2)	0 (0)		1 (1)	2 (1)	0.18
Neurological	2 (1)	0 (0)		1 (1)	1 (0)	0.74
Musculoskeletal	15 (4)	8 (6)		4 (3)	22 (6)	0.42
Diabetes	9 (2)	4 (3)		8 (6)	53 (14)	< 0.001
Any illness	57 (15)	19 (13)		21 (15)	85 (22)	0.016

*BMI* body mass index, *MET* metabolic equivalent, *LDL* low-density lipoprotein, *HDL* high-density lipoprotein

^a^Hommel's *p* < 0.05

### Cross-sectional associations between baseline body composition and baseline HRQoL

In the cross-sectional analyses, baseline FMI was negatively associated with several domains of HRQoL (adjusted for sex, age, education, smoking, alcohol consumption, physical activity, any severe long-term illness), i.e., general health (*β* = − 0.27, 95% confidence interval [95% CI] − 0.35 to − 0.18), physical functioning (*β* = − 0.38, 95% CI − 0.45 to − 0.30), role limitations caused by physical health problems (*β* = − 0.18, 95% CI − 0.27 to − 0.09), vitality (*β* = − 0.12, 95% CI − 0.21 to − 0.03), physical health component score (*β* = − 0.32, 95% CI − 0.40 to − 0.23), and bodily pain (*β* − 0.10, 95% CI − 0.19 to − 0.01) (Table 2, means for the four body composition categories in Table 3). Crude and adjusted model estimates differed only marginally, and, therefore, only adjusted estimates are shown. Higher lean mass index was associated with better general health (*β* = 0.13, 95% CI 0.03 to 0.23) and a higher physical component score (*β* = 0.13, 95% CI 0.03 to 0.22) at baseline. There were no clear indications that baseline body composition would be associated with baseline mental health, role limitations caused by mental health problems, social functioning or mental component score.

**Table 2:** Standardized regression coefficients of the adjusted linear regression analyses with continuous baseline fat mass index (FMI), lean mass index (LMI), and their interaction explaining health-related quality of life (HRQoL, RAND-36) domains at baseline

HRQoL baseline	Fat mass index		Lean mass index		FMI × LMI interaction	
	*β* (95% CI)	*p*	*β* (95% CI)	*p*	*β* (95% CI)	*p*
General health	− 0.27 (− 0.35 to − 0.18)	< 0.001	0.13 (0.03 to 0.23)	0.009	− 0.03 (− 0.10 to 0.03)	0.29
Physical functioning	− 0.38 (− 0.45 to − 0.30)	< 0.001	0.07 (− 0.02 to 0.17)	0.12	0.01 (− 0.06 to 0.06)	0.98
Role physical	− 0.18 (− 0.27 to − 0.09)	< 0.001	0.07 (− 0.04 to 0.17)	0.21	0.03 (− 0.03 to 0.10)	0.30
Bodily pain	− 0.10 (− 0.19 to − 0.01)	0.028	0.05 (− 0.05 to 0.16)	0.33	0.01 (− 0.05 to 0.08)	0.73
Mental health	0.01 (− 0.09 to 0.10)	0.90	0.01 (− 0.10 to 0.11)	0.88	− 0.04 (− 0.10 to 0.03)	0.25
Vitality	− 0.12 (− 0.21 to − 0.03)	0.007	0.08 (− 0.02 to 0.19)	0.12	− 0.06 (− 0.12 to 0.01)	0.089
Role emotional	0.01 (− 0.08 to 0.10)	0.81	− 0.08 (− 0.19 to 0.02)	0.12	− 0.04 (− 0.11 to 0.02)	0.20
Social functioning	− 0.06 (− 0.16 to 0.03)	0.18	0.05 (− 0.05 to 0.16)	0.32	0.03 (− 0.03 to 0.10)	0.31
Physical component score	− 0.32 (− 0.40 to − 0.23)	< 0.001	0.13 (0.03 to 0.22)	0.013	0.02 (− 0.04 to 0.08)	0.46
Mental component score	0.07 (− 0.03 to 0.16)	0.16	− 0.03 (− 0.13 to 0.08)	0.63	− 0.05 (− 0.11 to 0.02)	0.17

Independent variables included in the regression models: FMI, LMI, FMI × LMI interaction, sex, age, education, smoking, alcohol use, physical activity and severe long-term illness

The values of the standardized regression coefficients above 0.10, 0.30, and 0.50 represent small, moderate and large effect sizes, respectively

**Table 3:** Means (95% confidence intervals) of health-related quality of life (RAND-36) domains according to body composition categories

	Fat mass index low		Fat mass index high		*p* fat mass index	*p* lean mass index	*p* interaction
	Lean mass index low *N* = 377	Lean mass index high *N* = 144	Lean mass index low *N* = 377	Lean mass index high *N* = 144			
General health	69 (67–71)	68 (65–70)	64 (62–67)	65 (63–67)	< 0.001	0.61	0.31
Physical functioning	89 (88–90)	89 (87–91)	86 (84–88)	82 (81–84)	< 0.001	0.050	0.030
Role physical	88 (85–90)	88 (84–92)	84 (79–89)	81 (78–85)	0.009	0.51	0.49
Bodily pain	80 (78–83)	78 (75–82)	79 (75–83)	79 (77–81)	0.74	0.43	0.52
Mental health	82 (80–83)	82 (80–84)	82 (79–84)	82 (80–83)	0.97	0.96	0.94
Vitality	72 (70–74)	74 (71–77)	72 (69–75)	71 (69–73)	0.15	0.75	0.23
Role emotional	87 (84–90)	85 (81–90)	88 (83–92)	86 (83–88)	0.73	0.35	0.92
Social functioning	91 (89–93)	92 (89–94)	91 (89–94)	90 (89–92)	0.67	0.77	0.37
Physical component score	51 (50–52)	51 (49–52)	49 (48–50)	48 (47–50)	< 0.001	0.29	0.58
Mental component score	54 (53–55)	54 (53–55)	55 (53–56)	54 (54–55)	0.29	0.94	0.73

Models are adjusted for sex, age, education, smoking, alcohol use, physical activity, and severe long-term illness

*p*-values for the effect of fat mass index, lean mass index and their interaction

### Longitudinal associations between baseline body composition and change in HRQoL

**Fig. 2:**
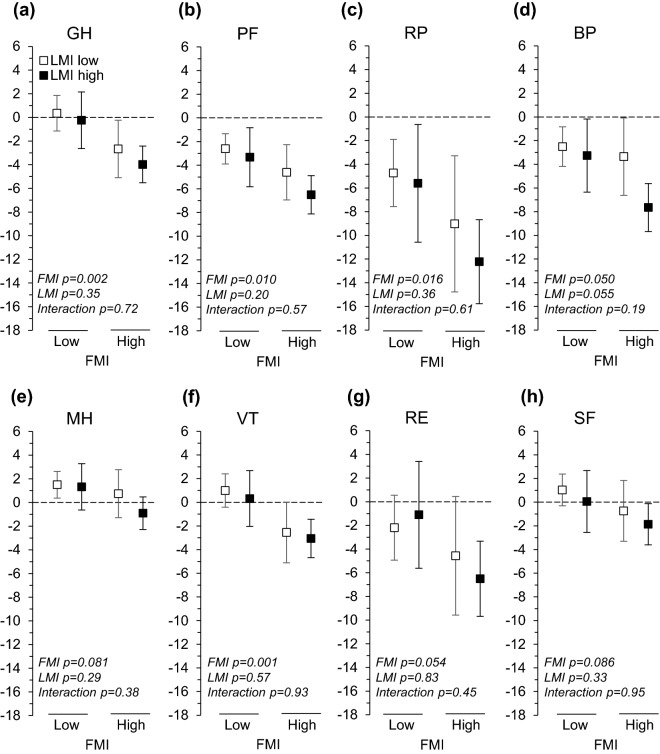
Adjusted changes in RAND-36 domains across body composition categories. ‘LMI low' refers to lean mass index (lean body mass/height^2^) equal or below the sex-specific median and ‘LMI high' to lean mass index above the sex-specific median. ‘FMI low' and ‘FMI high' are defined correspondingly. Models were adjusted for sex, age, education, smoking, alcohol consumption, physical activity, any severe long-term illness, and corresponding baseline RAND-36 domain. *GH* general health, *PF* physical functioning, *RP* role limitations caused by physical problems, *BP* bodily pain, *MH* mental health, *VT* vitality, *RE* role limitations caused by emotional problems, *SF* social functioning

**Fig. 3:**
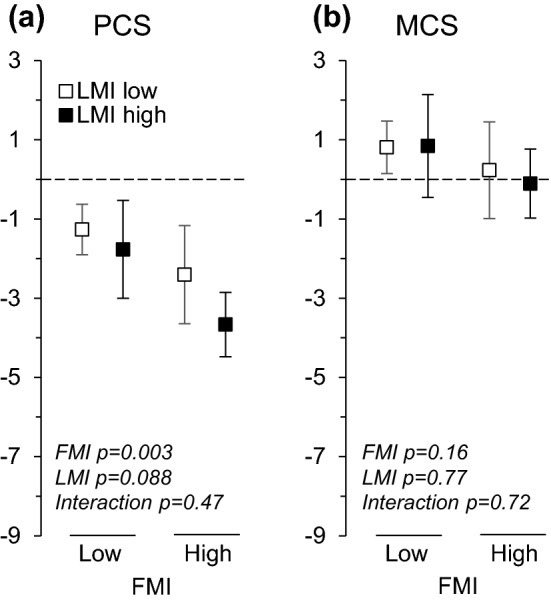
Adjusted changes in RAND-36 physical (PCS) and mental (MCS) component score across body composition categories. ‘LMI low' refers to lean mass index (lean body mass/height^2^) equal or below the sex-specific median and ‘LMI high' to lean mass index above the sex-specific median. ‘FMI low' and ‘FMI high' are defined correspondingly. Models were adjusted for sex, age, education, smoking, alcohol consumption, physical activity, any severe long-term illness, and corresponding baseline RAND-36 component score

There were no interactions of FMI and LMI on HRQoL variables. Adjusted for the baseline covariates (sex, age, education, smoking, alcohol consumption, physical activity, any severe long-term illness, and corresponding baseline HRQoL variable), higher FMI was associated with greater decline in general health (*β* = − 0.13, 95% CI − 0.20 to − 0.06), physical functioning (*β* = − 0.11, 95% CI − 0.18 to − 0.04), bodily pain (*β* = − 0.08, 95% CI − 0.16 to 0.01), role limitations caused by physical health problems (*β* = − 0.13, 95% CI − 0.22 to − 0.05), vitality (*β* = − 0.08, 95% CI − 0.16 to − 0.01), role limitations caused by emotional problems (*β* = − 0.12, 95% CI − 0.20 to − 0.03), and physical component score (*β* = − 0.14, 95% CI − 0.22 to − 0.07) (Table 4, means for the four body composition categories in Figs. 2 and 3). The changes in physical component score in the four body composition categories were the following: − 1.3 (LFLL), − 1.8 (LFHL), − 2.4 (HFLL), and − 3.7 (HFHL) points, respectively (Fig. 3). FMI was not associated with changes in the rest of the HRQoL measures. Lean mass index was not associated with changes in HRQoL measures.

**Table 4:** Standardized regression coefficients of adjusted linear regression analyses with continuous baseline fat mass index (FMI), lean mass index (LMI), and their interaction explaining changes in health-related quality of life (HRQoL, RAND-36) domains

HRQoL change	Fat mass index		Lean mass index		FMI × LMI interaction	
	*β* (95% CI)	*p*	*β* (95% CI)	*p*	*β* (95% CI)	*p*
General health	− 0.13 (− 0.20 to − 0.06)	0.001	− 0.01 (− 0.09 to 0.08)	0.90	− 0.01 (− 0.06 to 0.05)	0.84
Physical functioning	− 0.11 (− 0.18 to − 0.04)	0.002	− 0.01 (− 0.09 to 0.07)	0.80	− 0.01 (− 0.05 to 0.05)	0.94
Role physical	− 0.13 (− 0.22 to − 0.05)	0.003	0.01 (− 0.09 to 0.11)	0.82	0.01 (− 0.05 to 0.07)	0.67
Bodily pain	− 0.08 (− 0.16 to 0.01)	0.058	− 0.07 (− 0.17 to 0.02)	0.12	0.05 (− 0.01 to 0.11)	0.075
Mental health	− 0.02 (− 0.10 to 0.06)	0.57	− 0.07 (− 0.16 to 0.02)	0.15	− 0.03 (− 0.08 to 0.01)	0.33
Vitality	− 0.08 (− 0.16 to − 0.01)	0.027	− 0.04 (− 0.12 to 0.05)	0.37	0.02 (− 0.03 to 0.08)	0.41
Role emotional	− 0.12 (− 0.20 to − 0.03)	0.007	0.04 (− .06 to 0.14)	0.40	− 0.01 (− 0.06 to 0.06)	0.94
Social functioning	− 0.06 (− 0.14 to 0.03)	0.19	− 0.03 (− 0.13 to 0.07)	0.51	− 0.04 (− 0.10 to 0.02)	0.24
Physical component score	− 0.14 (− 0.22 to − 0.07)	< 0.001	− 0.02 (− 0.11 to 0.06)	0.58	0.02 (− 0.03 to 0.08)	0.43
Mental component score	− 0.03 (− 0.11 to 0.05)	0.49	− 0.02 (− 0.12 to 0.08)	0.68	− 0.03 (− 0.08 to 0.03)	0.40

Independent variables included in the regression models: FMI, LMI, FMI **×** LMI interaction, sex, age, education, smoking, alcohol use, physical activity, severe long-term illness, and corresponding HRQoL domain at baseline

The values of the standardized regression coefficients above 0.10, 0.30, and 0.50 represent small, moderate and large effect sizes, respectively

## Discussion

We examined the associations of body composition with HRQoL and its change during a 10 year period in older men and women simultaneously adjusting for fat and lean mass. Compared to those with low fat mass index, those with high fat mass index had lower scores in several physical health domains at baseline. They also showed larger declines in all physical health domains, and in the vitality and emotional role domains. Higher lean mass index was only associated with better general health and a higher physical component score at baseline.

Although several cross-sectional studies have examined the relationships between body composition and HRQoL in older adults [23–25, 40], only few have utilized a longitudinal design. In the present study, several domains of HRQoL declined in all four body composition categories over the 10-year follow-up. These were physical functioning, role limitations caused by physical health problems, and bodily pain. We observed mean declines of 1.3–3.7 points in the physical composite score (PCS) within the four body composition categories in the present study. A previous study reported a mean decline of about 4 points in PCS from the age 60 to 70 years, an age period comparable to that in the present study [41]. The largest declines were observed in role limitations caused by physical health problems but also the variance of change was largest in this domain.

Our findings are in agreement with a study reporting that a higher fat mass (assessed using dual-energy x-ray absorptiometry, DXA) was associated with a greater decline in SF-36 physical functioning domain over a 3-year follow-up among older women [26]. That study did not report other SF-36 domains. Further, they reported a U-shaped association between lean mass and change in physical functioning; those in the lowest and highest quartile of lean mass showed the greatest decline in physical functioning. When these analyses were adjusted for fat mass, the association of lean mass with physical functioning attenuated suggesting confounding by fat mass. However, most studies have ignored fat mass when studying lean mass and vice versa. A study including 65 years and older individuals reported no associations between change in a EQ-5D index over 3  years and a range of obesity measures, such as BMI, waist circumference, and fat percentage, although in cross-sectional analysis almost all measures were associated with HRQoL [27]. Another study explored HRQoL trajectories among over 4000 women, who were 60–79 years at baseline. The results suggested that obese women were more likely than others to be in a trajectory that had persistently low HRQoL index over the 7-year follow-up [42]. According to Cohen's reference values [39], the effect sizes for the associations between FMI and changes in HRQoL were generally small in the present study. Hence, there is some evidence suggesting that obesity is a risk factor for worsening physical HRQoL in older age.

Our cross-sectional findings on physical health domains across body composition categories are largely in line with previous studies, which have reported inverse cross-sectional associations between physical HRQoL and different indicators of obesity. BMI was negatively associated with the Short Form-36 (SF-36) physical component summary score and all physical health subdomains in a study including 205 adults aged 60 years and older [13]. Similar findings were reported in healthy women aged 45–70 years, but in healthy men in the same study, BMI was only associated with the physical functioning domain and physical component summary score [14]. Only few cross-sectional studies have used more sophisticated methods to assess adiposity. In a study among 40–75 year-old adults with hip or knee osteoarthritis, higher fat mass index, assessed by using DXA, was associated with lower SF-36 physical health summary score [40]. A population-based study among older men reported associations between SF-36 and multiple anthropometric/body composition measures. In that study, waist circumference was negatively associated with all physical SF-36 domains and most strongly with physical functioning [25]. Interestingly, waist circumference was superior to visceral adipose tissue area and subcutaneous adipose tissue area in explaining the variance in SF-36 scores. The authors speculated that the reason for this is that waist circumference combines the effects of visceral and subcutaneous fat. In our study, the differences between the high and low FMI category was 2.1 points for physical component score and 5.1 points for physical functioning, which reach the thresholds for minimal important differences [43]. Further, the effect sizes of the cross-sectional associations of physical functioning and physical component score with FMI were medium, while those of general health, role physical, bodily pain, and vitality with FMI were small.

Findings on the associations between lean mass and HRQoL seem to be more inconsistent than those between adiposity and HRQoL. We found that lean mass index was positively, but weakly associated with general health and the physical component score, but not with other HRQoL domains, in the analyses using continuous lean mass index. Glintborg et al. reported no associations between absolute lean body mass and SF-36 domains in older men [25]. Furthermore, in older adults with hip or knee osteoarthritis, fat-free mass index or appendicular lean mass (ALM) to BMI ratio were not significantly associated with SF-36 physical component summary score [40]. However, low muscle mass was associated with a general HRQoL index, EQ-5D index score, as well as mobility, self-care, and usual activities dimensions of EQ-5D among older Korean men [24]. Among Korean women, low muscle mass was not associated with HRQoL dimensions after controlling for the covariates, including BMI. This study suggested that lower cut-off points for ALM discriminate better between those with low and high HRQoL than higher cut-off points. Hence, the relationship between lean mass and HRQoL may not be linear but may have a certain threshold below which lean mass has a negative effect on HRQoL. Muscle strength may be more important than muscle mass for HRQoL, since muscle strength has been reported to have larger effect sizes than lean mass on HRQoL [23]. This may be caused by the faster age-related decline in muscle strength than in muscle mass [23].

We found that body composition was not strongly associated with mental HRQoL. Baseline fat mass index explained changes only in domains assessing vitality and emotional role. Some of the previous cross-sectional studies have reported no associations between BMI and mental health domains of the SF-36 [14] or SF-12 mental component score [44]. However, waist circumference was reported to be inversely associated with all SF-36 mental domains among older Danish men [25]. Further, a study comparing obese older adults and non-obese, non-frail older adults reported that obese older adults had lower scores in mental HRQoL than non-obese non-frail older adults [45]. However, only the difference in vitality reached statistical significance. Jeanmaire et al. reported a positive association between appendicular lean mass to BMI ratio and SF-36 mental composite score in older adults with hip or knee osteoarthritis [40]. However, fat-free mass index or indicators of fat mass were not associated with mental HRQoL. Unlike HRQoL related to physical health, HRQoL related to mental health was largely maintained at the same level over the 10-year follow-up in the present study. This is in agreement with previous studies suggesting that mental HRQoL does not decline markedly, if at all, in older age [41, 46, 47].

Mechanisms linking physical HRQoL and its change to increased fat mass may include an array of diseases. Obesity is a well-known risk factor for several non-communicable diseases, such as cardiovascular diseases, several cancers, and diabetes, all prevalent in older age [48]. The emergence of these diseases may lead to decline in the domains of physical HRQoL [49]. We adjusted for diseases that are likely to have a major impact on HRQoL but it is plausible that undiagnosed and less severe illnesses, not captured by our disease variable, explain at least some of the association between fat mass and HRQoL. Increased fat mass may also hamper physical functioning directly by augmenting mechanical load in everyday activities. In addition, obese older adults are less physically active than older adults with normal weight [50], which may have caused poorer physical functioning and consequently resulted in lower HRQoL in those with high fat mass. On the other hand, it is also possible that an increased fat mass index is a consequence of decline in HRQoL. Older adults who have problems with functioning tend to be less physically active [51], which may, in turn, increase fat mass over time. Nevertheless, we found a prospective association between baseline fat mass index and change in HRQoL, which suggests that at least some of the association is explained by a link directed from fat mass to physical HRQoL.

A strength of this study is that we simultaneously took into account both fat and lean mass, which may have a mutual confounding effect on HRQoL; further, we also studied the interaction of fat and lean mass. The study sample was a community-derived sample, which was followed up for 10 years enabling analyzing changes in HRQoL. We analyzed both physical and mental domains of HRQoL. This study has some limitations. Use of DXA in assessing body composition would have ensured better validity [52]. However, bioelectrical impedance analysis correlates well with DXA at the group level [52] but its validity may be lower among older adults e.g., due to edema. Information on lifestyle variables, used as covariates, were obtained using questionnaires and only at baseline. This may have caused residual confounding. A characteristic feature of studies consisting of older adults, including the present study, is that there is a considerable loss of participants in the follow-up. This loss was not completely random but those who participated in the follow-up had better functioning at baseline than those who did not participate in the follow-up [53] and hence, these results may be applicable only to older adults with relatively good functional status. Our participants were Caucasian and the results may, therefore, not be generalizable to other populations.

## Conclusion

A high fat mass in older age, independent of lean mass, threatens physical HRQoL but mental HRQoL is less affected by high fat mass. Prevention of adiposity in older adults may contribute to a good quality of life in older age.
